# (*E*)-2-[(4-Chloro-1,3-dimethyl-1*H*-pyrazol-5-yl)methyl­eneamino]benzamide

**DOI:** 10.1107/S1600536809050685

**Published:** 2009-11-28

**Authors:** Yunbo Cong, Huibin Yang, Haibo Yu, Bin Li

**Affiliations:** a Shenyang Institute of Chemical Technology, Shenyang 110142, People’s Republic of China; b Agrochemicals Division, Shenyang Research Institute of Chemical Industry, Shenyang 110021, People’s Republic of China

## Abstract

In the title compound, C_13_H_13_ClN_4_O, the dihedral angle between the aromatic rings is 33.47 (9)° and an intra­molecular N—H⋯N hydrogen bond generates an *S*(6) ring. In the crystal, inversion dimers linked by pairs of N—H⋯O hydrogen bonds occur, resulting in *R*_2_^2^(8) loops.

## Related literature

For catalytic studies on a related compound, see: Chen *et al.* (2008 ▶).
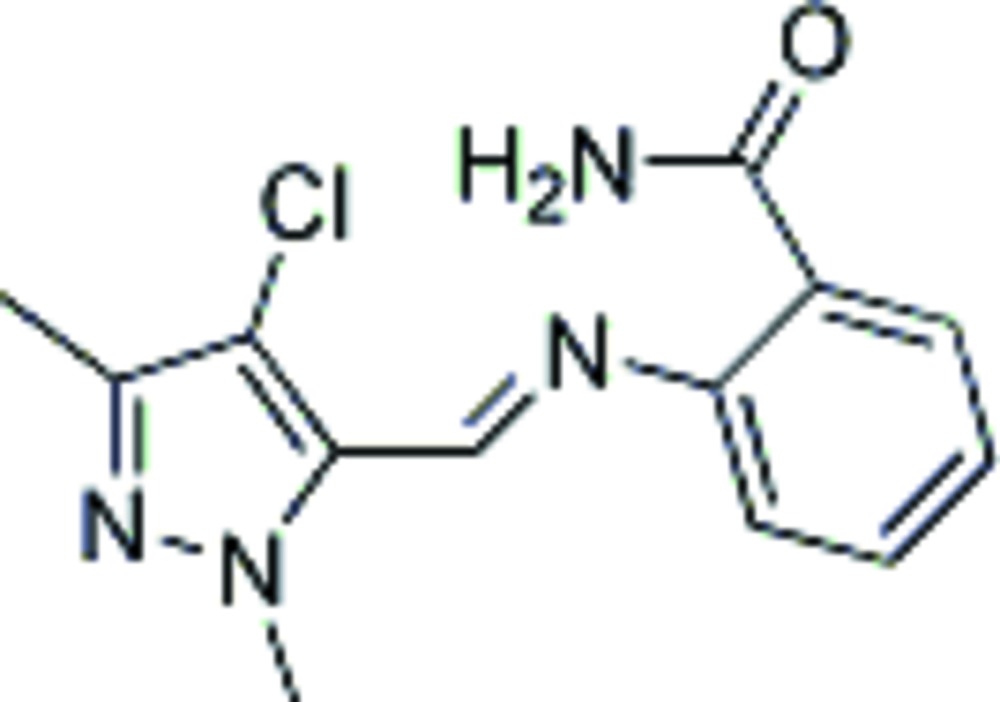


## Experimental

### 

#### Crystal data

C_13_H_13_ClN_4_O
*M* *_r_* = 276.72
Monoclinic, 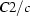

*a* = 22.046 (3) Å
*b* = 8.6785 (10) Å
*c* = 14.1137 (16) Å
β = 96.005 (2)°
*V* = 2685.5 (5) Å^3^
*Z* = 8
Mo *K*α radiation
μ = 0.28 mm^−1^
*T* = 296 K
0.32 × 0.30 × 0.28 mm

#### Data collection

Bruker SMART CCD diffractometer
Absorption correction: multi-scan (*SADABS*; Bruker, 2001 ▶) *T*_min_ = 0.725, *T*_max_ = 1.000
6634 measured reflections
2375 independent reflections
2098 reflections with *I* > 2σ(*I*)
*R*_int_ = 0.018

#### Refinement

*R*[*F* ^2^ > 2σ(*F* ^2^)] = 0.035
*wR*(*F* ^2^) = 0.098
*S* = 1.06
2375 reflections
175 parameters
H-atom parameters constrained
Δρ_max_ = 0.17 e Å^−3^
Δρ_min_ = −0.19 e Å^−3^

### 

Data collection: *SMART* (Bruker, 2001 ▶); cell refinement: *SAINT* (Bruker, 2001 ▶); data reduction: *SAINT*; program(s) used to solve structure: *SHELXS97* (Sheldrick, 2008 ▶); program(s) used to refine structure: *SHELXL97* (Sheldrick, 2008 ▶); molecular graphics: *SHELXTL* (Sheldrick, 2008 ▶); software used to prepare material for publication: *SHELXTL*.

## Supplementary Material

Crystal structure: contains datablocks I, global. DOI: 10.1107/S1600536809050685/hb5252sup1.cif

Structure factors: contains datablocks I. DOI: 10.1107/S1600536809050685/hb5252Isup2.hkl

Additional supplementary materials:  crystallographic information; 3D view; checkCIF report

## Figures and Tables

**Table 1:** Hydrogen-bond geometry (Å, °)

*D*—H⋯*A*	*D*—H	H⋯*A*	*D*⋯*A*	*D*—H⋯*A*
N4—H4*B*⋯N3	0.86	2.07	2.749 (2)	136
N4—H4*A*⋯O1^i^	0.86	2.10	2.930 (2)	161

Symmetry code: (i) 
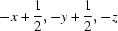
.

**Fig. 1.**
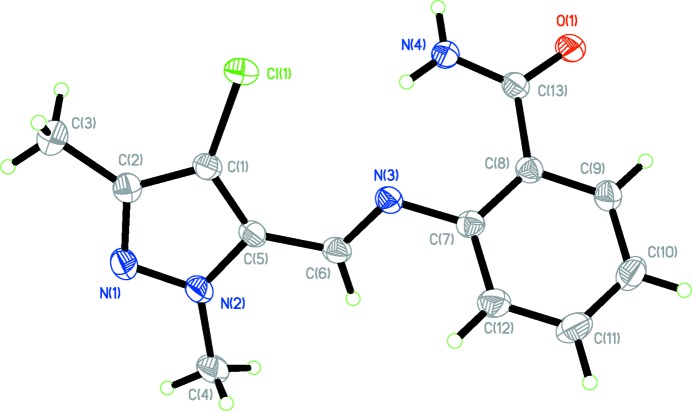
The molecular structure of (I), with 30% probability displacement ellipsoids.

## supplementary crystallographic information

### Experimental

The title compound was synthesized by the reaction of 4-chloro-1,3-dimethyl-1*H*-pyrazole-5- carbaldehyde with 2-aminobenzamide in 1,2-dichloroethane. The crude product was purified by silica-gel column chromatography and then grown from methylene chloride to afford colourless blocks of (I).

Anal. Calcd for C_13_H_13_N_4_O_1_: C, 56.42; H, 4.74; N, 20.25; O, 5.78. Found: C, 56.57; H, 4.64; N, 20.66; O, 5.25. ^1^H NMR(DMSO): 2.21(s,3*H*, CH_3_), 4.12 (s,3*H*, N—CH_3_), 7.33(s, 2H, CONH_2_), 7.50–7.61 (m, 2H, Ph), 7.89 (t, 1H, Ph—H), 8.26 (s, 1H, Ph—H), 8.46 (s, 1H, CH=N).

### Refinement

All H atoms were visible in difference maps; they were finally placed in geometrically calculated positions, with C—H = 0.93–0.96Å and N—H = 0.86 Å, and refined as riding with *U*_iso_(H) = 1.2*U*_eq_(C, N) or 1.5*U*_eq_(methyl C).

### Crystal data

**Table:**

C_13_H_13_ClN_4_O	*F*(000) = 1152
*M**_r_* = 276.72	*D*_x_ = 1.369 Mg m^−^^3^
Monoclinic, *C*2/*c*	Mo *K*α radiation, λ = 0.71073 Å
Hall symbol: -C 2yc	Cell parameters from 4144 reflections
*a* = 22.046 (3) Å	θ = 2.2–30.2°
*b* = 8.6785 (10) Å	µ = 0.28 mm^−^^1^
*c* = 14.1137 (16) Å	*T* = 296 K
β = 96.005 (2)°	Block, colourless
*V* = 2685.5 (5) Å^3^	0.32 × 0.30 × 0.28 mm
*Z* = 8	

### Data collection

**Table:**

Bruker SMART CCD diffractometer	2375 independent reflections
Radiation source: fine-focus sealed tube	2098 reflections with *I* > 2σ(*I*)
graphite	*R*_int_ = 0.018
ω scans	θ_max_ = 25.0°, θ_min_ = 1.9°
Absorption correction: multi-scan (*SADABS*; Bruker, 2001)	*h* = −25→26
*T*_min_ = 0.725, *T*_max_ = 1.000	*k* = −6→10
6634 measured reflections	*l* = −16→16

### Refinement

**Table:**

Refinement on *F*^2^	Secondary atom site location: difference Fourier map
Least-squares matrix: full	Hydrogen site location: inferred from neighbouring sites
*R*[*F*^2^ > 2σ(*F*^2^)] = 0.035	H-atom parameters constrained
*wR*(*F*^2^) = 0.098	*w* = 1/[σ^2^(*F*_o_^2^) + (0.052*P*)^2^ + 1.4091*P*] where *P* = (*F*_o_^2^ + 2*F*_c_^2^)/3
*S* = 1.06	(Δ/σ)_max_ < 0.001
2375 reflections	Δρ_max_ = 0.17 e Å^−^^3^
175 parameters	Δρ_min_ = −0.19 e Å^−^^3^
0 restraints	Extinction correction: *SHELXL97* (Sheldrick, 2008), Fc^*^=kFc[1+0.001xFc^2^λ^3^/sin(2θ)]^-1/4^
Primary atom site location: structure-invariant direct methods	Extinction coefficient: 0.0266 (12)

### Special details

**Table:**

Geometry. All e.s.d.'s (except the e.s.d. in the dihedral angle between two l.s. planes) are estimated using the full covariance matrix. The cell e.s.d.'s are taken into account individually in the estimation of e.s.d.'s in distances, angles and torsion angles; correlations between e.s.d.'s in cell parameters are only used when they are defined by crystal symmetry. An approximate (isotropic) treatment of cell e.s.d.'s is used for estimating e.s.d.'s involving l.s. planes.

### Fractional atomic coordinates and isotropic or equivalent isotropic displacement parameters (Å^2^)

**Table:**

	*x*	*y*	*z*	*U*_iso_*/*U*_eq_	
Cl1	0.45129 (2)	0.49955 (4)	0.12551 (4)	0.0612 (2)	
N4	0.29251 (6)	0.43146 (17)	0.04310 (11)	0.0531 (4)	
H4A	0.3006	0.3480	0.0140	0.064*	
H4B	0.3217	0.4899	0.0670	0.064*	
O1	0.19254 (5)	0.38809 (14)	0.01737 (10)	0.0600 (4)	
N1	0.52045 (6)	0.91074 (16)	0.12334 (10)	0.0486 (4)	
N2	0.46079 (6)	0.94242 (16)	0.12143 (10)	0.0445 (3)	
C6	0.36052 (7)	0.82150 (17)	0.12489 (11)	0.0397 (4)	
H6	0.3417	0.9174	0.1202	0.048*	
N3	0.32795 (6)	0.70280 (14)	0.13302 (9)	0.0376 (3)	
C1	0.46698 (7)	0.69287 (17)	0.12368 (11)	0.0400 (4)	
C2	0.52480 (7)	0.75722 (19)	0.12428 (11)	0.0430 (4)	
C3	0.58468 (8)	0.6779 (2)	0.12766 (15)	0.0576 (5)	
H3A	0.6159	0.7518	0.1181	0.086*	
H3B	0.5831	0.6013	0.0784	0.086*	
H3C	0.5938	0.6294	0.1886	0.086*	
C4	0.44200 (9)	1.1028 (2)	0.12241 (16)	0.0614 (5)	
H4C	0.4316	1.1292	0.1847	0.092*	
H4D	0.4072	1.1179	0.0766	0.092*	
H4E	0.4749	1.1672	0.1066	0.092*	
C5	0.42578 (7)	0.81350 (17)	0.12263 (11)	0.0379 (4)	
C7	0.26516 (7)	0.72357 (17)	0.14214 (10)	0.0364 (3)	
C8	0.22185 (7)	0.61479 (17)	0.10333 (10)	0.0363 (3)	
C9	0.16068 (7)	0.6395 (2)	0.11507 (12)	0.0445 (4)	
H9	0.1317	0.5694	0.0890	0.053*	
C10	0.14195 (8)	0.7653 (2)	0.16439 (13)	0.0541 (5)	
H10	0.1007	0.7807	0.1701	0.065*	
C11	0.18475 (8)	0.8675 (2)	0.20506 (14)	0.0577 (5)	
H11	0.1726	0.9509	0.2398	0.069*	
C12	0.24531 (8)	0.8468 (2)	0.19446 (13)	0.0504 (4)	
H12	0.2738	0.9163	0.2227	0.060*	
C13	0.23540 (7)	0.46978 (18)	0.05100 (11)	0.0401 (4)	

### Atomic displacement parameters (Å^2^)

**Table:**

	*U*^11^	*U*^22^	*U*^33^	*U*^12^	*U*^13^	*U*^23^
Cl1	0.0557 (3)	0.0314 (3)	0.0956 (4)	−0.00049 (17)	0.0037 (2)	−0.0028 (2)
N4	0.0376 (7)	0.0445 (8)	0.0774 (10)	−0.0038 (6)	0.0064 (7)	−0.0244 (7)
O1	0.0391 (6)	0.0426 (7)	0.0960 (10)	−0.0043 (5)	−0.0039 (6)	−0.0204 (6)
N1	0.0387 (7)	0.0446 (8)	0.0618 (9)	−0.0076 (6)	0.0021 (6)	0.0017 (6)
N2	0.0407 (7)	0.0328 (7)	0.0589 (8)	−0.0046 (5)	−0.0004 (6)	0.0008 (6)
C6	0.0404 (8)	0.0316 (8)	0.0452 (8)	0.0002 (6)	−0.0044 (6)	0.0003 (6)
N3	0.0356 (7)	0.0345 (7)	0.0418 (7)	−0.0026 (5)	−0.0006 (5)	−0.0032 (5)
C1	0.0417 (8)	0.0332 (8)	0.0442 (8)	−0.0017 (6)	−0.0001 (6)	−0.0015 (6)
C2	0.0403 (8)	0.0434 (9)	0.0450 (9)	−0.0012 (7)	0.0025 (7)	−0.0004 (7)
C3	0.0447 (10)	0.0609 (12)	0.0674 (12)	0.0047 (8)	0.0066 (8)	0.0000 (9)
C4	0.0561 (11)	0.0322 (9)	0.0946 (15)	−0.0052 (8)	0.0017 (10)	−0.0010 (9)
C5	0.0389 (8)	0.0322 (8)	0.0414 (8)	−0.0043 (6)	−0.0018 (6)	−0.0007 (6)
C7	0.0374 (8)	0.0346 (8)	0.0364 (7)	0.0027 (6)	0.0004 (6)	0.0009 (6)
C8	0.0367 (8)	0.0338 (8)	0.0381 (8)	−0.0006 (6)	0.0018 (6)	0.0023 (6)
C9	0.0366 (8)	0.0452 (9)	0.0513 (9)	−0.0019 (7)	0.0024 (7)	0.0001 (7)
C10	0.0404 (9)	0.0613 (11)	0.0614 (11)	0.0102 (8)	0.0097 (8)	−0.0044 (9)
C11	0.0547 (11)	0.0574 (11)	0.0608 (11)	0.0142 (9)	0.0046 (8)	−0.0175 (9)
C12	0.0478 (10)	0.0461 (9)	0.0557 (10)	0.0014 (8)	−0.0012 (8)	−0.0145 (8)
C13	0.0379 (8)	0.0343 (8)	0.0472 (8)	−0.0027 (6)	0.0009 (6)	−0.0010 (6)

### Geometric parameters (Å, °)

**Table:**

Cl1—C1	1.7137 (16)	C3—H3B	0.9600
N4—C13	1.318 (2)	C3—H3C	0.9600
N4—H4A	0.8600	C4—H4C	0.9600
N4—H4B	0.8600	C4—H4D	0.9600
O1—C13	1.2356 (18)	C4—H4E	0.9600
N1—C2	1.336 (2)	C7—C12	1.396 (2)
N1—N2	1.3413 (19)	C7—C8	1.412 (2)
N2—C5	1.360 (2)	C8—C9	1.392 (2)
N2—C4	1.452 (2)	C8—C13	1.505 (2)
C6—N3	1.2677 (19)	C9—C10	1.381 (2)
C6—C5	1.444 (2)	C9—H9	0.9300
C6—H6	0.9300	C10—C11	1.376 (3)
N3—C7	1.4151 (19)	C10—H10	0.9300
C1—C5	1.385 (2)	C11—C12	1.371 (2)
C1—C2	1.391 (2)	C11—H11	0.9300
C2—C3	1.485 (2)	C12—H12	0.9300
C3—H3A	0.9600		
			
C13—N4—H4A	120.0	H4C—C4—H4E	109.5
C13—N4—H4B	120.0	H4D—C4—H4E	109.5
H4A—N4—H4B	120.0	N2—C5—C1	104.45 (14)
C2—N1—N2	105.89 (13)	N2—C5—C6	121.91 (13)
N1—N2—C5	112.81 (13)	C1—C5—C6	133.62 (14)
N1—N2—C4	118.44 (13)	C12—C7—C8	118.74 (14)
C5—N2—C4	128.70 (14)	C12—C7—N3	120.63 (13)
N3—C6—C5	122.55 (14)	C8—C7—N3	120.52 (13)
N3—C6—H6	118.7	C9—C8—C7	118.35 (14)
C5—C6—H6	118.7	C9—C8—C13	115.57 (13)
C6—N3—C7	118.26 (13)	C7—C8—C13	126.07 (13)
C5—C1—C2	107.22 (14)	C10—C9—C8	121.76 (16)
C5—C1—Cl1	127.38 (12)	C10—C9—H9	119.1
C2—C1—Cl1	125.40 (13)	C8—C9—H9	119.1
N1—C2—C1	109.62 (14)	C11—C10—C9	119.45 (16)
N1—C2—C3	121.67 (15)	C11—C10—H10	120.3
C1—C2—C3	128.70 (16)	C9—C10—H10	120.3
C2—C3—H3A	109.5	C12—C11—C10	120.20 (16)
C2—C3—H3B	109.5	C12—C11—H11	119.9
H3A—C3—H3B	109.5	C10—C11—H11	119.9
C2—C3—H3C	109.5	C11—C12—C7	121.41 (16)
H3A—C3—H3C	109.5	C11—C12—H12	119.3
H3B—C3—H3C	109.5	C7—C12—H12	119.3
N2—C4—H4C	109.5	O1—C13—N4	121.44 (15)
N2—C4—H4D	109.5	O1—C13—C8	119.04 (14)
H4C—C4—H4D	109.5	N4—C13—C8	119.52 (13)
N2—C4—H4E	109.5		
			
C2—N1—N2—C5	0.99 (18)	N3—C6—C5—C1	3.3 (3)
C2—N1—N2—C4	178.50 (16)	C6—N3—C7—C12	−37.2 (2)
C5—C6—N3—C7	175.07 (13)	C6—N3—C7—C8	146.70 (15)
N2—N1—C2—C1	−0.46 (18)	C12—C7—C8—C9	3.1 (2)
N2—N1—C2—C3	−179.23 (15)	N3—C7—C8—C9	179.31 (13)
C5—C1—C2—N1	−0.19 (18)	C12—C7—C8—C13	−175.57 (15)
Cl1—C1—C2—N1	−179.43 (12)	N3—C7—C8—C13	0.6 (2)
C5—C1—C2—C3	178.46 (17)	C7—C8—C9—C10	−0.9 (2)
Cl1—C1—C2—C3	−0.8 (3)	C13—C8—C9—C10	177.86 (15)
N1—N2—C5—C1	−1.09 (18)	C8—C9—C10—C11	−1.5 (3)
C4—N2—C5—C1	−178.29 (17)	C9—C10—C11—C12	1.7 (3)
N1—N2—C5—C6	177.42 (13)	C10—C11—C12—C7	0.5 (3)
C4—N2—C5—C6	0.2 (3)	C8—C7—C12—C11	−2.9 (3)
C2—C1—C5—N2	0.75 (17)	N3—C7—C12—C11	−179.15 (16)
Cl1—C1—C5—N2	179.96 (12)	C9—C8—C13—O1	4.2 (2)
C2—C1—C5—C6	−177.51 (16)	C7—C8—C13—O1	−177.06 (15)
Cl1—C1—C5—C6	1.7 (3)	C9—C8—C13—N4	−174.85 (15)
N3—C6—C5—N2	−174.72 (15)	C7—C8—C13—N4	3.9 (2)

### Hydrogen-bond geometry (Å, °)

**Table:**

*D*—H···*A*	*D*—H	H···*A*	*D*···*A*	*D*—H···*A*
N4—H4B···N3	0.86	2.07	2.749 (2)	136
N4—H4A···O1^i^	0.86	2.10	2.930 (2)	161

Symmetry codes: (i) −*x*+1/2, −*y*+1/2, −*z*.
